# Rural residence and mental health among US Veterans: Findings from the Millennium Cohort Study

**DOI:** 10.1371/journal.pone.0346780

**Published:** 2026-04-17

**Authors:** Claire A. Kolaja, Javier Villalobos, Julia Seay, Hope S. McMaster, Edward J. Boyko, Rudolph P. Rull, for the Millennium Cohort Study Team

**Affiliations:** 1 Deployment Health Research Department, Naval Health Research Center, San Diego, California, United States of America; 2 Leidos, Inc., San Diego, California, United States of America; 3 Seattle Epidemiologic Research and Information Center, VA Puget Sound Healthcare System, Seattle, Washington, United States of America; 4 Department of Medicine, University of Washington School of Medicine, Seattle, Washington, United States of America; Georgia Southern University, UNITED STATES OF AMERICA

## Abstract

**Background:**

Military veterans are at higher risk of mental health conditions compared to civilians. Some residential characteristics may increase the odds of mental health conditions, but other studies have not examined location attributes (i.e., residential rurality, community social vulnerability, and drive time from Veterans Health Administration [VHA] facilities) and VHA utilization concurrently in association with veteran mental health.

**Methods:**

We analyzed data from 20,423 veterans enrolled in the Millennium Cohort Study, the longest running and largest study of service members and veterans, who completed one pre- and one post-military separation survey (Time 1 and 2, respectively). Time 2 mailing addresses were used to determine location characteristics, including rurality, social vulnerability, and drive time to VHA facilities. VHA enrollment/utilization and mental health outcomes, including probable PTSD, depression, anxiety, and mental quality of life (QOL), were also measured at Time 2. Models adjusting for Time 1 military, demographic and behavioral characteristics were used to examine the association of location characteristics and VHA enrollment/utilization on mental health.

**Results:**

Rurality was not associated with the four mental health outcomes examined. VHA utilization was associated with elevated odds of probable PTSD, depression, anxiety, and lower mental QOL. While community social vulnerability trended toward associations with PTSD and depression, it was only statistically associated with higher odds of anxiety.

**Discussion:**

This study was able to examine residential attributes and VHA utilization on post military mental health. In adjusted models, we found that rurality and drive time to VHA facilities were not associated with mental health. The association between VHA enrollment and utilization with worse mental health was consistent and strong in all models examined.

## Introduction

Military veterans are at a higher risk of mental health conditions [1] than the general United States population. The transition from military to civilian life can be a period of intense stress, with challenges from culture shock, interpersonal problems, and employment difficulties potentially increasing this risk [2–4]. The socio-ecological model, which posits that mental health is influenced by a combination of individual attributes and the social and physical environment, provides a robust framework for understanding these dynamics [5]. Within this model, psychological health is shaped by factors across multiple levels—individual, interpersonal, community, and societal—that collectively contribute to both risk and resilience [5].

Prior work suggests that contextual factors may affect mental health as well as individual characteristics like sex, age, or race [6,7]. Environmental features such as green space, walkability, and the presence or absence of physical disorder (e.g., graffiti, traffic) can either mitigate or exasperate stress [8]. In a systematic literature review, certain indicators of neighborhood safety were found to be consistently associated with mental health [9]. Sources of danger can increase feelings of vulnerability while decreasing perceived control, which can result in psychological stress [10,11]. Being the victim or witnessing a crime specifically can increase psychological responses [12]. Areas with high crime rates also have less social cohesion and higher social disorganization [13]. Social isolation can impact feelings of belonging and perceived social support, while sociocultural factors like stoicism and stigma can also vary geographically [14]. Finally, the concentration of healthcare providers in metropolitan areas in the US may hinder access to services and delay treatment for those living elsewhere [15].

To our knowledge, no study examining veteran mental health has explicitly framed their hypotheses using the socio-ecological model, though some have examined related indicators. When examining neighborhood resources and mental health, Park et al. (2021) found that living in alcohol-permissive and tobacco-restrictive communities was associated with psychological distress, while urban/rural residence and access to facilities were not [16]. Another study found that neighborhoods with the lowest socioeconomic status experienced worse mental quality of life [17].

Rurality is one of the most frequently examined contextual factors in veteran mental health literature, often serving as a proxy for individual and community-level sociodemographic factors like lower income and education levels, as well as isolation and fewer resources. However, research examining the mental health of veterans by rurality (e.g., urban versus rural areas) has yielded inconsistent results, often because it fails to account for location attributes. For example, a cross-sectional study of 767,109 VHA-enrolled veterans found that those living in rural areas had worse mental quality of life as determined by the Medical Outcomes Study Short-form 36 (SF-36) Mental Component Summary (MCS) scores than those living in urban settings [18]. Conversely, another study of VHA-enrolled veterans found a higher prevalence of psychiatric disorders among veterans residing in urban areas, yet rural veterans with these disorders experienced worse overall mental health [19]. In contrast, a recent longitudinal analysis of veterans using VHA outpatient services found that those in rural areas reported better overall mental health, although the rural-urban difference decreased over time [20]. Finally, a study examining veterans in the Millennium Cohort Study did not find any differences between those living in rural and urban areas (n = 10,738) [21].

The heterogeneity of these findings may be accounted for by differences in other location attributes. Contextual factors like crime, economic condition, and healthcare access are not randomly distributed and often co-occur. Therefore, it is preferable to use a composite measure that combines several of these factors. Social vulnerability, or the social conditions that affect vulnerability to adverse events such as natural disasters and diseases, may vary widely both between and within urban and rural areas. The U.S. Census created the Social Vulnerability Index (SVI) to measure the susceptibility of communities based on community factors such as poverty, age, transportation, and housing characteristics. Among post-disaster civilian populations, regions with higher SVI scores (indicating more vulnerability) had worse mental and physical health outcomes [22]. Similarly, longer drive times to VHA providers may be an indicator of vulnerability for veterans and reduced access to services. Results from civilian studies regarding the impact of distance to health care facilities on mental health have been mixed [23], which may be due to inconsistencies in study designs and measures of distance. Although the type of rurality (isolated vs micropolitan) and variability in other location attributes may influence veteran mental health, prior studies have not comprehensively examined these factors simultaneously.

Another limitation of prior research examining health differences by urban-rural location is the reliance on overly simplistic definitions of rurality [24]. Most studies have used discrete categories (e.g., urban vs rural) based on population size, density, or commuting patterns. These definitions often fail to differentiate between larger, “micropolitan” rural areas (nonmetropolitan counties with populations between 10,000 and 50,000) and smaller rural areas that are considered “isolated” (defined in the current study by Rural Urban Community Area [RUCA] codes). This distinction may be salient, as some studies have found that individuals living in isolated rural areas may have poorer access to mental healthcare providers as well as higher levels of loneliness than those living in micropolitan rural areas, which may in turn influence mental health outcomes [25,26].

Therefore, there is a clear need to examine the relationship between rurality and mental health among veterans with greater specificity, not only to better understand the potential influence of location on veteran mental health, but also to inform future interventions to address mental health risk and resource allocation among veterans. By not restricting our sample to veterans within the VHA network (i.e., utilizing VHA services), the current study overcame a key limitation of previous research and allowed us to examine whether the influence of residential location on mental health conditions varied by VHA healthcare **enrollment** as well as by VHA healthcare **utilization**. Mental health problems (depression, PTSD, anxiety) and mental QOL were compared among veterans enrolled in the Millennium Cohort Study [27] living in isolated rural, micropolitan rural, and urban areas. Our analyses also incorporated social vulnerability and drive time from VHA facilities to clarify the complex associations between residential location and mental health.

## Methods

### Study population

The current study analyzed data from the Millennium Cohort Study, the largest and longest-running population-based study of service members. A detailed description of the study methods has been published elsewhere [27]. Briefly, participants were enrolled while serving in the military and then surveyed every 3–5 years, both during service and after military separation. The first panel of service members was enrolled in 2001 with additional panels enrolled in 2004, 2007, 2011, and 2020. To date, over 260,000 participants representing all service branches and components are enrolled across these five panels. Participants provided voluntary, informed consent at enrollment on the self-administered web or paper survey and the study protocol was approved by the Naval Health Research Center institutional review board.

In this nested cross-sectional study, Panel 1–4 participants (enrolled between July 1, 2001 and April 4, 2013) were eligible for these analyses if they completed at least one survey while actively serving in the military as active duty, Reserve or National Guard (Time 1) and one survey after separating from the military (Time 2; n = 45,418). Furthermore, as location attributes (i.e., rurality, social vulnerability, and drive time from VHA facilities) were of interest in this study, participants were excluded if their Time 2 location was not in the United States or was not known (n = 12,224). Participants missing Time 2 mental health outcome (n = 5,686), military discharge status (n = 2,049), or Time 1 covariate information (n = 2,582) were excluded from analyses. Further exclusions included completion of the Time 2 survey during the first fiscal year participants were observed in the VHA data (n = 2,454), as exact timing (i.e., before or after Time 2 survey) could not be determined from available annual VHA enrollment and utilization data. This resulted in a final study sample of 20,423 participants.

### Measures

#### Mental health outcomes.

The four mental health outcomes (i.e., PTSD, depression, anxiety, overall mental QOL) were measured at Time 2 (i.e., the first survey completed after separating from the military). PTSD was measured using the 17-item Posttraumatic Stress Disorder Checklist – Civilian Version (PCL-C). PTSD screening criteria were consistent with diagnostic criteria of the Diagnostic and Statistical Manual of Mental Disorders, 4^th^ Edition, Text Revision (DSM-IV-TR) of endorsing “Moderately” or higher on at least 1 intrusion item, 2 hyper-arousal items, and 3 avoidance items [28–30]. Probable major depressive disorder (MDD) was measured using the 8-item Patient Health Questionnaire (PHQ-8) consistent with the DSM-IV-TR criteria of endorsing at least 5 items as “More than half the days” or higher, in which 1 of the symptoms was anhedonia or depressed mood [31,32]. Symptoms of “panic syndrome” and “other anxiety syndrome” in the previous 4 weeks were assessed using criteria for 2 subscales of the PHQ [33]. “Other anxiety syndrome” was indicated if participants reported “More than half the days” to “Feeling nervous, anxious, on edge, or worrying a lot about different things” and at least 3 of the 6 remaining items on the subscale. Probable “panic syndrome” was indicated if all 4 items about feeling sudden fear/panic were endorsed, as well as to at least 4 of the 11 follow-up symptom items. Subscales were combined so that screening positive for either anxiety or panic syndrome indicated probable anxiety/panic (henceforth referred to as “anxiety”). Finally, overall mental QOL was measured with the mental component score from the Short Form 36 – Health Survey for Veterans (SF-36V), which combines all eight subscales while weighting the mental health scales more heavily. Mental QOL was treated as a continuous outcome, with a mean of 50 and standard deviation of 10, and with higher scores indicating better mental QOL [34].

#### Health care utilization.

Utilization of VHA through September 2019 was ascertained from a VHA-constructed dataset that provided information by fiscal year regarding participant enrollment and/or utilization of VHA healthcare services. These annual indicators were compared to the Time 2 survey date to categorize participants as 1) not enrolled in the VHA, 2) enrolled but had not utilized VHA care, and 3) enrolled and utilized VHA care by completion of the Time 2 survey.

#### Residential location characteristics.

Participants’ mailing addresses during Time 2 survey completion were geocoded using the Streetmap Premium extension in ArcGIS Pro (ESRI, 2023) and used to assess three residential location attributes of interest. First, we defined rurality based on the RUCA classification system which was developed by the Washington, Wyoming, Alaska, Montana, Idaho (WWAMI) Rural Health Research Center (RHRC) [35] using population estimates and percent of the community commuting to urban areas. For the current analyses, Categorization B was used to identify veterans residing in urban (RUCA codes 1, 1.1, 2, 2.1, 3, 4.1, 5.1, 7.1, 8.1, 10.1), micropolitan rural (RUCA codes 4, 4.2, 5, 5.2, 6, 6.1), and isolated rural (RUCA codes 7, 7.2, 7.3, 7.4, 8, 8.2, 8.3, 8.4, 9, 9.1, 9.2, 10, 10.2, 10.3, 10.4, 10.5, 10.6) regions. To assess the influence of the operationalization of rurality, we conducted post-hoc analyses replacing discrete RUCA codes with the continuous Index of Relative Rurality (IRR), which combines four indicators: population size, density, remoteness, and built-up area (see supplemental materials for additional details).

Additionally, the residential address of each veteran when they completed their Time 2 survey was assigned a score for social vulnerability using the Centers for Disease Control and Prevention’s (CDC’s) Social Vulnerability Index (SVI). A higher overall percentile ranking for a community indicates relatively more vulnerability based on the four main themes: socioeconomic, household composition/disability, minority status/language, and housing type/transportation [36]. For the purposes of this study, the percentile was standardized with a mean of zero and a standard deviation of one.

Finally, drive time to VHA facilities was determined using Time 2 participant mailing addresses and VHA facilities addresses from the Medicare Wage Index [37]. ArcGIS Pro mapping software was used to identify residences that were more than a 30-minute drive time from the closest VHA healthcare facility. This threshold was chosen to reflect the eligibility requirement from the 2019 VA MISSION Act that expanded access to community healthcare to eligible veterans.

#### Covariates.

Demographic and military characteristics (age, race, ethnicity, pay grade, service branch, military occupation) were obtained from the Defense Manpower Data Center (DMDC) and if time varying, represent the status as of the Time 1 survey. Type of military discharge condition and date of military separation was also obtained from DMDC and used to represent type of military separation and time between military separation and Time 2 survey completion. Educational attainment and marital status were self-reported on the Time 1 survey and backfilled with DMDC data if self-report information was missing. Enrollment panel was also included as a covariate to account for potential effect measure heterogeneity.

Deployments before the Time 1 survey were identified based on electronic military deployment data in support of operations in Iraq and Afghanistan obtained from DMDC and were combined with combat experience(s) self-reported on the Time 1 survey using 5 items (e.g., “witnessing a person’s death due to war, disaster, or tragic event,” “witnessing instances of physical abuse, such as torture, beating, rape”). DMDC deployment history and combat survey items were used to classify veterans as 1) not deployed, 2) deployed with no combat, or 3) deployed with combat experience [38].

Other covariates, such as problematic drinking, smoking status, sleep duration, physical QOL, mental health status (i.e., probable PTSD, MDD, anxiety and mental QOL), were assessed using Time 1 survey data. Problematic drinking was assessed using the 5-item alcohol abuse and dependence module of the PHQ which assesses symptoms of alcohol misuse over the past 12 months (e.g., “You drove a car after having several drinks or after drinking too much,” “You drank alcohol even though a doctor suggested that you stop drinking because of a problem with your health”). A positive screen for problematic drinking was registered if a yes response was given to one or more items [33]. Smoking status was determined using lifetime report of smoking at least 100 cigarettes and successfully quitting into: never smoker (had not smoked at least 100 cigarettes), former smoker (smoked at least 100 cigarettes and successfully quit) and current smoker (smoked at least 100 cigarettes and had not successfully quit) [39]. Sleep duration was based on a single survey item measuring the average number of hours slept in an average 24-hour period over the past month. Participants who reported 7–9 hours of sleep were considered to sleep the recommended sleep duration [40]. Physical QOL was assessed using the physical component summary score from the Veterans Short Form-36 (SF-36V), with higher scores indicating better overall physical health [34]. Time 1 mental health was assessed using the same methods described above for the outcomes of interest and included as a covariate in the adjusted model for each outcome (e.g., Time 1 probable PTSD was adjusted for in the model Time 2 probable PTSD).

### Statistical analysis

Descriptive and bivariate analyses compared residential location rurality with mental health outcomes of interest, VHA health care utilization, other location characteristics, and covariates. Next, to examine the associations between VHA utilization and location characteristics (rurality, social vulnerability, drive time to VHA facilities) with mental health outcomes, logistic regression models were fit to predict probable depression, PTSD, and anxiety, and linear regression models were fit to predict mental QOL. First, unadjusted associations between predictors with mental health outcomes were estimated. Then, models adjusting for panel, age, sex, race, ethnicity, educational attainment, marital status, military characteristics (i.e., pay grade, service branch, military occupation, deployment experience, discharge condition, years since military separation), problem drinking, smoking status, PCS, sleep duration, and Time 1 mental health (PTSD, depression, anxiety, mental QOL) were generated for each outcome. To investigate our secondary aim of determining whether relationships differ between rurality by health care utilization, an interaction term for rurality and VHA care utilization was included in each fully adjusted model. Multicollinearity was assessed using the variance inflation factor, in which a value of 4 or greater indicated possible collinearity. All analyses were conducted using SAS/STAT software, version 9.4.

## Results

### Descriptive results

Among the 20,423 veterans eligible for these analyses, 88.3% lived in an urban location, 7.6% lived in a micropolitan rural location, and 4.1% lived in an isolated rural location at Time 2 (Table 1). Overall, Time 2 prevalence of probable PTSD was 16.8%, depression was 11.4%, anxiety was 13.4%, and the average mental QOL was 47.2 (standard deviation [SD]: 12.8). More than half (52.0%) of participants had enrolled in *and* utilized VHA care before the Time 2 survey, while 5.2% enrolled but *did not* utilize VHA care, and 42.7% had *not contacted* the VHA before the Time 2 survey.

**Table 1:** Characteristics of mental health outcomes and predictors of interest by rurality± among Veteran Millennium Cohort Study participants.

	Study Sample n = 20,423	Urban N = 18,047 (88.3%)	Micropolitan rural N = 1,545 (7.6%)	Isolated rural N = 831 (4.1%)	p-value
**Mental Health Outcome**±					
Probable PTSD	3,433 (16.8)	2,984 (16.5)	288 (18.6)	161 (19.4)	0.02
Probable Depression	2,355 (11.4)	2,037 (11.3)	182 (11.8)	116 (14.0)	0.06
Probable Anxiety	2,741 (13.4)	2,394 (13.3)	224 (14.5)	123 (14.8)	0.19
Mental QOL (mean, SD)	47.2 (12.8)	47.2 (12.8)	46.9 (13.0)	46.5 (13.0)	0.15
**Healthcare Utilization**±					
VHA utilization					0.01
Not enrolled	8,724 (42.7)	7,786 (43.1)	615 (39.8)	323 (38.9)	
Enrolled, did not utilize	1,072 (5.2)	953 (5.3)	81 (5.2)	38 (4.6)	
Enrolled and utilized	10,627 (52.0)	9,308 (51.6)	849 (55.0)	470 (56.6)	
**Other Location Attributes**±					
Social Vulnerability Index overall percentile, higher percentiles are relatively more vulnerable (mean, SD)	0.4 (0.3)	0.4 (0.3)	0.5 (0.2)	0.6 (0.2)	<.0001
Address within a 30-minute drive to VHA facility	17,948 (87.9)	16,554 (91.7)	1,049 (67.9)	345 (41.5)	<.0001
**Demographics+**					
Age (mean, SD)	31.5 (8.8)	31.6 (8.8)	30.8 (8.7)	29.6 (8.3)	<.0001
Sex					0.01
Male	14,105 (69.1)	12,407 (68.7)	1,118 (72.4)	580 (69.8)	
Female	6,318 (30.9)	5,640 (31.3)	427 (27.6)	251 (30.2)	
Race and ethnicity					<.0001
American Indian	284 (1.4)	243 (1.3)	26 (1.7)	15 (1.8)	
Asian or Pacific Islander	747 (3.7)	716 (4.0)	17 (1.1)	14 (1.7)	
Black non-Hispanic	2,258 (11.1)	2,146 (11.9)	80 (5.2)	32 (3.9)	
Hispanic/Latino	1,499 (7.3)	1,389 (7.7)	74 (4.8)	36 (4.3)	
Other	255 (1.2)	236 (1.3)	12 (0.8)	7 (0.8)	
White non-Hispanic	15,380 (75.3)	13,317 (73.8)	1,336 (86.5)	727 (87.5)	
Educational attainment					<.0001
High school or less	3,912 (19.2)	3,344 (18.5)	335 (21.7)	233 (28.0)	
Some college	8,655 (42.4)	7,539 (41.8)	735 (47.6)	381 (45.8)	
Associate degree	2,711 (13.3)	2,404 (13.3)	216 (14.0)	91 (11.0)	
Bachelor’s degree	3,035 (14.9)	2,790 (15.5)	159 (10.3)	86 (10.3)	
Graduate degree	2,110 (10.3)	1,970 (10.9)	100 (6.5)	40 (4.8)	
Marital status					0.01
Single, never married	6,102 (29.9)	5,358 (29.7)	463 (30.0)	281 (33.8)	
Married	11,905 (58.3)	10,514 (58.3)	931 (60.3)	460 (55.4)	
Previously married	2,416 (11.8)	2,175 (12.1)	151 (9.8)	90 (10.8)	
**Military Characteristics+**					
Military rank					<.0001
Junior enlisted	8,995 (44.0)	7,810 (43.3)	747 (48.3)	438 (52.7)	
Noncommissioned officer	8,222 (40.3)	7,257 (40.2)	636 (41.2)	329 (39.6)	
Officer	3,206 (15.7)	2,980 (16.5)	162 (10.5)	64 (7.7)	
Service branch					0.22
Army	7,848 (38.4)	6,878 (38.1)	637 (41.2)	333 (40.1)	
Navy or Coast Guard	4,276 (20.9)	3,804 (21.1)	297 (19.2)	175 (21.1)	
Marine Corps	2,649 (13.0)	2,343 (13.0)	202 (13.1)	104 (12.5)	
Air Force	5,650 (27.7)	5,022 (27.8)	409 (26.5)	219 (26.4)	
Deployment experience					0.04
Not deployed	9,710 (47.5)	8,642 (47.9)	701 (45.4)	367 (44.2)	
Deployed, no combat	4,222 (20.7)	3,729 (20.7)	323 (20.9)	170 (20.5)	
Deployed with combat	6,491 (31.8)	5,676 (31.5)	521 (33.7)	294 (35.4)	
Military occupation					0.47
Admin or supply	5,639 (27.6)	5,000 (27.7)	412 (26.7)	227 (27.3)	
Healthcare	2,269 (11.1)	2,022 (11.2)	154 (10.0)	93 (11.2)	
Other	9,131 (44.7)	8,046 (44.6)	702 (45.4)	383 (46.1)	
Combat specialist	3,384 (16.6)	2,979 (16.5)	277 (17.9)	128 (15.4)	
Discharge condition					
Honorable	15,375 (75.3)	13,590 (75.3)	1,175 (76.1)	610 (73.4)	0.01
General, honorable conditions	335 (1.6)	279 (1.5)	30 (1.9)	26 (3.1)	
Bad, dishonorable, or other than honorable	163 (0.8)	137 (0.8)	15 (1.0)	11 (1.3)	
Reservist or National Guardsmen	4,550 (22.3)	4,041 (22.4)	325 (21.0)	184 (22.1)	
Years separated from military service (mean, SD)	3.1 (2.1)	3.0 (2.1)	3.1 (2.1)	3.3 (2.2)	0.01
**Behavioral/Physical/Mental Health+**					
Problem drinking	2,641 (12.9)	2,314 (12.8)	200 (12.9)	127 (15.3)	0.12
Smoking status					<.0001
Never smoked	11,199 (54.8)	10,096 (55.9)	759 (49.1)	344 (41.4)	
Former smoker	5,162 (25.3)	4,498 (24.9)	418 (27.1)	246 (29.6)	
Current smoker	4,062 (19.9)	3,453 (19.1)	368 (23.8)	241 (29.0)	
Sleeps recommended duration (7–9 hrs)	7,710 (37.8)	6,866 (38.0)	558 (36.1)	286 (34.4)	0.04
Physical QOL (mean, SD)	51.4 (9.0)	51.4 (9.0)	51.0 (9.3)	51.4 (8.8)	0.16
Mental QOL (mean, SD)	49.3 (11.0)	49.4 (11.0)	49.1 (10.8)	48.1 (11.7)	<0.01
Probable depression	1,390 (6.8)	1,216 (6.7)	110 (7.1)	64 (7.7)	0.49
Probable PTSD	1,956 (9.6)	1,727 (9.6)	140 (9.1)	89 (10.7)	0.43
Probable anxiety	1,490 (7.3)	1,301 (7.2)	115 (7.4)	74 (8.9)	0.18

**+**measured at Time 1; ± measured at Time 2; PTSD, posttraumatic stress disorder; QOL, quality of life; SD, standard deviation; VHA, Veterans Health Administration.

The average age of participants at Time 1 was 31.5 years (SD: 8.8 years) and a majority were male (69.1%) and non-Hispanic White (75.3%). At Time 1, the most common characteristics were some college experience but had not obtained a degree (42.4%), married (58.3%), junior enlisted pay grade (44.0%), serving in the Army (38.4%), had not deployed (47.5%), and worked in other occupations (e.g., electrical or mechanical equipment repair, craft workers; 44.7%). At separation, most were discharged honorably (75.3%) with an average of 3.1 years (SD: 2.1) between separation from the military and the completion of the Time 2 survey. Most participants did not screen positive for problem drinking (87.1%), never smoked cigarettes (54.8%), did not screen positive for depression, PTSD, and anxiety (93.2%, 90.4%, 92.7%, respectively), and slept between 7 and 9 hours (62.2%). Average physical QOL of 51.4 (SD: 9.0) and mental QOL of 49.3 (SD: 11.0) were observed at Time 1.

### Bivariate associations

At the bivariate level, rurality was associated with probable PTSD and not associated with depression, anxiety, or mental QOL (Table 2). That is, compared to veterans in urban locations, those in micropolitan rural or isolated rural locations had higher odds of PTSD (micropolitan odds ratios [OR], 95% confidence interval [95% CI]: 1.16, 1.01–1.32 and isolated OR, 95% CI: 1.21, 1.02–1.45). VHA utilization was associated with all mental health outcomes, such that, compared to those *not enrolled* in the VHA by Time 2, veterans who had *enrolled and not utilized* care were more likely to screen positive for PTSD (OR, 95% CI: 1.42, 1.15–1.75), depression (OR, 95% CI: 1.34, 1.03–1.73), anxiety (OR, 95% CI: 1.55, 1.23–1.95) and report lower mental QOL (mean difference −1.02, standard error [SE]: 0.40). Furthermore, the association for veterans who *enrolled and utilized* VHA care was even stronger; they were more likely to screen positive for PTSD (OR 3.99, 95% CI: 3.65–4.37), depression (OR 3.83, 95% CI: 3.44–4.26), anxiety (OR 4.05, 95% CI: 3.66–4.48), and report lower mental QOL (mean difference −6.14, SE: 0.18). Social vulnerability was also associated with all four mental health outcomes; a one standard deviation increment in community social vulnerability (SVI) was associated with greater odds of screening positive for PTSD (OR 1.22, 95% CI: 1.18–1.27), depression (OR 1.21, 95% CI: 1.16–1.27), anxiety (OR 1.20, 95% CI: 1.15–1.25), and lower mental QOL (mean difference −1.11, SE: 0.09). Longer drive times to VHA facilities was not associated with any of the outcomes.

**Table 2:** Regression models examining relationship between rurality, health care utilization, and other location attributes on mental health among Veteran Millennium Cohort Study participants (n = 20,423).

	Unadjusted		Adjusted±	
	OR (95% CI)	p-value	AOR (95% CI)	p-value
Probable PTSD				
Rurality (ref: urban)		0.01		0.45
Micropolitan rural	1.16 (1.01, 1.32)		1.11 (0.95, 1.29)	
Noncore rural	1.21 (1.02, 1.45)		1.03 (0.84, 1.28)	
VHA utilization (ref: never enrolled)		<.0001		<.0001
Enrolled, did not utilize	1.42 (1.15, 1.75)		1.17 (0.93, 1.46)	
Enrolled and utilized	3.99 (3.65, 4.37)		2.61 (2.35, 2.90)	
Social Vulnerability Index+	1.22 (1.18, 1.27)	<.0001	1.04 (1.00, 1.09)	0.08
More than 30 mins to VHA Facility (ref: within 30 minutes)	1.01 (0.91, 1.13)	0.82	0.93 (0.81, 1.07)	0.30
Probable depression				
Rurality (ref: urban)		0.06		0.65
Micropolitan rural	1.05 (0.89, 1.23)		0.95 (0.79, 1.14)	
Noncore rural	1.28 (1.04, 1.56)		1.08 (0.85, 1.36)	
VHA utilization (ref: never enrolled)		<.0001		<.0001
Enrolled, did not utilize	1.34 (1.03, 1.73)		1.12 (0.85, 1.46)	
Enrolled and utilized	3.83 (3.44, 4.26)		2.56 (2.26, 2.88)	
Social Vulnerability Index+	1.21 (1.16, 1.27)	<.0001	1.04 (0.99, 1.10)	0.08
More than 30 mins to VHA Facility (ref: within 30 minutes)	1.05 (0.92, 1.19)	0.50	1.01 (0.87, 1.18)	0.90
Probable anxiety				
Rurality (ref: urban)		0.19		0.55
Micropolitan rural	1.11 (0.96, 1.29)		0.98 (0.83, 1.16)	
Noncore rural	1.14 (0.93, 1.38)		0.88 (0.70, 1.11)	
VHA utilization (ref: never enrolled)		<.0001		<.0001
Enrolled, did not utilize	1.55 (1.23, 1.95)		1.27 (1.00, 1.63)	
Enrolled and utilized	4.05 (3.66, 4.48)		2.66 (2.38, 2.98)	
Social Vulnerability Index+	1.20 (1.15, 1.25)	<.0001	1.06 (1.01, 1.11)	0.02
More than 30 mins to VHA Facility (ref: within 30 minutes)	1.04 (0.92, 1.17)	0.58	1.01 (0.87, 1.16)	0.95
Mental QOL				
	Estimate (SE)		Estimate (SE)*	
Rurality (ref: urban)		0.15		0.06
Micropolitan rural	−0.35 (0.34)		0.40 (0.29)	
Noncore rural	−0.78 (0.46)		0.83 (0.40)	
VHA utilization (ref: never enrolled)		<.0001		<.0001
Enrolled, did not utilize	−1.02 (0.40)		0.23 (0.34)	
Enrolled and utilized	−6.14 (0.18)		−3.01 (0.17)	
Social Vulnerability Index+	−1.11 (0.09)	<.0001	−0.13 (0.08)	0.09
More than 30 mins to VHA Facility (ref: within 30 minutes)	0.01 (0.28)	0.98	0.12 (0.24)	0.61

**±**Models adjusted for panel, age, sex, race and ethnicity, education attainment, marital status, pay grade, service branch, military occupation, deployment experience, discharge status, problem drinking, smoking status, physical components summary score, mental health, sleep duration, and years since separation. All interactions examined between rurality and VHA utilization were not statistically significant (*p* > 0.05). PTSD, posttraumatic stress disorder; QOL, quality of life; VHA, Veterans Health Administration; OR, odds ratio; AOR, adjusted odds ratio. + Estimates for social vulnerability index represent a standardized deviation change in score. Associations for the variables included in the adjusted models in Table 2 are displayed in S1 Table.

### Adjusted associations

In the adjusted models (Table 2), rurality did not appear to be associated with the mental health outcomes of interest. VHA utilization was significantly associated with all outcomes; veterans who *enrolled and utilized* VHA care by Time 2 showed a greater than twofold difference in screening positive for PTSD (adjusted odds ratio; AOR 2.61, 95% CI: 2.35–2.90), depression (AOR 2.56, 95% CI: 2.26–2.88), and anxiety (AOR 2.66, 95% CI: 2.38–2.98), and reported lower mental QOL (adjusted mean difference −3.01, SE: 0.17) compared to veterans not enrolled. Greater community social vulnerability was significantly associated with higher odds of anxiety (AOR 1.06, 95% CI: 1.01–1.11), while positive associations between community social vulnerability with PTSD and depression trended towards significance. Drive time to VHA facilities was not associated with any of the mental health outcomes. Finally, we investigated if mental health varied by rurality and VHA utilization by including the interaction between rurality and VHA utilization in the adjusted model for each outcome; interactions did not meet the specified statistical significance threshold (*p*-value > .05). Associations for the covariates included in the adjusted models in Table 2 and mental health outcomes are displayed in S1 Table.

### Supplemental analyses

See supplemental material for results from the post-hoc analyses conducted utilizing a continuous measure of rurality (Index of Relative Rurality [IRR]). In brief, bivariate and adjusted association between continuous rurality and the mental health outcomes examined aligned with those reported for the main analyses. One notable difference was found for the mental QOL in the fully adjusted model; IRR was significantly associated with mental QOL (*p-value* = 0.03) such that for a one unit increase in IRR, mental QOL was 1.54 units higher (S2 Table).

## Discussion

The World Health Organization (WHO) emphasizes that mental health is shaped not only by individual attributes but also by the social circumstances and environment in which individuals live [41]. This perspective is rooted in the socio-ecological model, which is broadly used to conceptualize psychological health and to identify factors at different levels (individual, interpersonal, community, and society) that may contribute to risk and resiliency [5]. Although several studies have examined associations between rurality and veteran health, to our knowledge, no studies have concurrently examined additional community attributes, such as social vulnerability, and drive time to VHA facilities, alongside VHA utilization, in a sample that includes both VHA-enrolled and non VHA-enrolled veterans. This study aimed to examine the relationship between residential location and veteran mental health using the broad socio-ecological framework to help disentangle the conflicting findings reported in previous studies. Final analyses indicated that veterans enrolled in *and* utilizing VHA care were more likely to screen positive for mental health conditions. Rurality was associated only with mental QOL, where *micropolitan rural and isolated rural* veterans reported modestly higher mental QOL compared with those in urban areas. Longer drive time to VHA facilities was not associated with post-military separation mental health.

Previous literature on rurality and veteran mental health reported inconsistent findings [18,20,21,42,43] that may be artifacts of heterogeneous definitions of rurality and mental health outcomes examined, the specific geographic location (e.g., a single state), examined older data, or focused on veterans enrolled in or utilizing VHA care. Descriptive results from the current study revealed that compared to urban counterparts, veterans living in isolated locations were more likely to be younger, less educated, junior enlisted pay grade at Time 1, experienced a deployment with combat, and discharged from the military with general or bad, dishonorable, or other than honorable status. Many of these are known risk factors for mental health conditions [44], which may explain why, when compared to veterans living in urban areas, veterans living in isolated rural locations were more likely to have PTSD in bivariate analyses, but no association between rurality and PTSD was observed in the adjusted model. While prior research has theorized that heightened isolation or a lack of resources in rural areas may negatively impact health [16,19,42,43,45–49], certain veterans who select rural residence may prefer various aspects of rural residence (e.g., privacy, peace, and quiet) [50] that may support and improve mental health. One notable finding from the adjusted models supportive of this theory was that veterans living in rural locations had modestly higher (better) mental QOL. This association reached statistical significance in the supplemental analyses using the continuous rurality measure. To our knowledge, no other study on veteran mental health has utilized a continuous measure of rurality although prior work conducted among civilians has compared continuous and categorical measures and highlighted similar findings (e.g., that the statistical significance mostly remained consistent regardless of rurality measure used) [51].

Regardless of how it is operationalized, rurality is a proxy for more specific location-based attributes. To investigate the socio-ecological model more directly, this study included additional community characteristics, such as social vulnerability and drive time to VHA facilities. While the associations between social vulnerability with PTSD, depression and mental QOL (*p-values* = 0.08–0.09) did not reach statistical significance, this marginal trend may suggest that living in more socially vulnerable areas has an adverse impact on mental health. This interpretation aligns with civilian studies reporting relationships between greater social vulnerability and worse mental health outcomes [22,52], potentially due to factors like fewer community resources [16] and heightened safety concerns [53]. Our nuanced findings are consistent with the broader literature. Prior meta-analyses and systematic reviews also reported heterogenous results when linking neighborhood factors to individual mental health [54,55]. This underscores that the detected relationships are often sensitive to the specific sample and methods used, highlighting the complexity of the socio-ecological model.

Furthermore, we found that residing more than a 30-minute drive from VHA healthcare facilities was not associated with mental health outcomes. While previous studies of veterans utilizing VHA care identified distance as a primary barrier to healthcare access [56,57], our finding aligns with contemporary research and may reflect a new reality in veteran healthcare [16]. The expansion of healthcare coverage for veterans through the CHOICE Act of 2014 and the VHA’s significant investment in telehealth infrastructure [44,45] may have fundamentally changed how veterans access services, potentially mitigating the historical impact of geographic distance [46–48].

In the fully adjusted models, we consistently found a positive association between utilization of VHA for healthcare with screening positive for mental health conditions. This finding aligns with several other studies that similarly found that VHA utilizers report worse mental health than veterans not utilizing VHA care [58–62]. This is not surprising as eligibility for VHA enrollment and prioritization of enrollment is based on service-related factors and income criteria [63], which skews the enrolled population to patients with higher health care needs. These findings align with the integration of mental health care in VHA primary care, resulting in more patients being screened and treated. These programs emphasize early detection and intervention of mental health symptoms. Prior work found that over 60% of veterans who screen positive for PTSD in a primary care setting engage with mental health treatment afterward (e.g., referral to a mental health clinic) [64]. The VHA may benefit from future research on barriers (contextual and individual-level) that hinder mental healthcare receipt among veterans.

The limitations of the current study should be considered when interpreting the findings described above. The present study leveraged self-reported data using mental health screeners, and therefore findings regarding mental health outcomes do not specifically refer to clinical diagnoses. As almost half of participants did not utilize the VHA for healthcare (42.7% were not enrolled and 5.2% were enrolled but did not utilize care), it was not feasible to incorporate clinical diagnosis of mental health conditions in this study. Actual diagnosis at the VHA by rurality is a separate analytic question and warrants future study. The study was also missing information on healthcare received outside of the VHA. Outcomes were reported between 2004–2016 and could have been influenced by external factors, such as global and economic events, that may not be accounted for in these models. The rurality categorization used in this study differed from the classification utilized by the VHA Office of Rural Health (ORH), which may limit the ability to compare findings. The classification used by the VA ORH categorizes even fewer veterans in isolated areas, however, post-hoc analyses revealed that using this categorization did not change the reported findings (screening positive for PTSD *p* = 0.50, major depression *p* = 0.74, and anxiety/panic *p* = 0.92, and MCS *p* = 0.08). In addition, as mailing addresses were used to determine rurality, there is a chance that residence could be different for some participants. We were not able to examine the full spectrum of individual (e.g., birth and childhood location, number of moves) and built environment factors that may vary by rurality that could affect mental health, such as available green space, noise from road traffic, and pollution. Finally, the Millennium Cohort Study enrolled service members beginning in July of 2001. Therefore, the results we report may not apply to veterans prior to this time.

This study has multiple notable strengths. The Millennium Cohort Study is the largest and longest running cohort study of service members and designed to follow participants after military separation through follow-up surveys and VHA records. Mailing addresses were merged with location specific factors (rurality, social vulnerability, drive time to VHA facilities) so that these factors could be explored concurrently in adjusted models. Finally, we were able to examine all veterans enrolled in the Millennium Cohort Study and not restricted to those utilizing VHA care.

In conclusion, this study explored associations between post-military location attributes and VHA utilization with veteran mental health while controlling for pre-separation characteristics among a relatively young sample of post 9/11 veterans within a few years of separation. Rurality and drive time to VHA facilities were not associated with mental health conditions after adjustment for pertinent covariates. Instead, VHA enrollment and utilization were consistently associated with worse mental health. These findings highlight the association between VHA enrollment and utilization with mental health symptoms and may contribute to VHA understanding of veterans with higher mental health needs as well as resource allocation.

## Supporting information

S1 Table Associations between risk and protective factors with Time 2 mental health outcomes among Veteran Millennium Cohort Study participants as included in adjusted models in Table 2, n = 20,423. (DOCX)

S2 Table Associations between continuous rurality and mental health outcomes, n = 20,418. (DOCX)
