# Supplementary material for: Rural residence and mental health among US Veterans: Findings from the Millennium Cohort Study
Source: PLoS One. 2026 Apr 17;21(4):e0346780. doi: 10.1371/journal.pone.0346780 (PMC13089753; doi:10.1371/journal.pone.0346780)
Supplement: S1 Table — (DOCX) [file pone.0346780.s001.docx]

**Rural residence and mental health among US Veterans:**

**Findings from the Millennium Cohort Study**

Claire A. Kolaja, Javier Villalobos, Julia Seay, Hope S. McMaster, Edward J. Boyko, Rudolph P. Rull, for the Millennium Cohort Study Team

PLOS One

**Associations between Covariates and Mental Health Outcomes**

In the interest of full transparency, we have added Supplemental Table 1, which details the results for all covariates from our main adjusted models. Age, marital status, panel, service branch, deployment experience, discharge condition, smoking status, sleep duration, physical QOL, and prior mental health were associated with all four mental health outcomes examined. Sex was only associated with mental QOL; race and ethnicity was associated with anxiety and mental QOL; education attainment was associated with probable PTSD and anxiety; pay grade was associated with PTSD and depression; military occupation was associated with probable PTSD, depression and mental QOL; and problem drinking was associated with probable PTSD, depression, and anxiety. Years since military separation was not associated with any of the mental outcomes. All associations were in the expected direction and align with prior publications. For example, the following factors were associated with higher odds of screening positive for mental health conditions: younger age, lower pay grade, having deployed with combat, general honorable or bad, dishonorable or other than honorable military discharge, former or current smoking, not reporting recommended sleep duration, and prior mental health status.

| **Supplemental Table 1: Associations between risk and protective factors with Time 2 mental health outcomes among Veteran Millennium Cohort Study participants as included in adjusted models in Table 2, n=20,423** | | | | |
| --- | --- | --- | --- | --- |
|  | **Probable**  **PTSD** | **Probable Depression** | **Probable Anxiety** | **Mental QOL** |
|  | OR (95% CI) | OR (95% CI) | OR (95% CI) | Coefficient (SE) |
| **Demographics at Time 1** | | | | |
| Age | 0.99 (0.98, 1.00) | 0.99 (0.98, 1.00) | 0.99 (0.98, 1.00) | 0.12 (0.02) |
| Female sex (ref: male) | 1.05 (0.95, 1.17) | 1.09 (0.97, 1.22) | 1.35 (1.21, 1.50) | -0.72 (0.18) |
| Race and ethnicity (ref: white non-Hispanic) |  |  |  |  |
| American Indian | 1.41 (1.03, 1.93) | 1.44 (1.02, 2.04) | 1.14 (0.81, 1.60) | -0.13 (0.64) |
| Asian or Pacific Islander | 1.07 (0.85, 1.34) | 1.19 (0.93, 1.52) | 0.76 (0.59, 0.99) | 0.46 (0.40) |
| Black non-Hispanic | 1.08 (0.94, 1.25) | 1.10 (0.94, 1.29) | 0.86 (0.74, 1.01) | 0.68 (0.25) |
| Hispanic or Latino | 1.18 (1.01, 1.38) | 1.20 (1.01, 1.43) | 1.19 (1.01, 1.40) | 0.09 (0.29) |
| Other | 1.28 (0.88, 1.84) | 1.19 (0.78, 1.81) | 0.74 (0.47, 1.18) | -0.62 (0.67) |
| Educational attainment (ref: graduate degree) |  |  |  |  |
| High school or less | 1.42 (1.06, 1.89) | 1.16 (0.85, 1.59) | 1.15 (0.85, 1.56) | -0.48 (0.44) |
| Some college | 1.52 (1.16, 1.98) | 1.11 (0.83, 1.49) | 1.31 (0.99, 1.74) | -0.50 (0.40) |
| Associate degree | 1.44 (1.09, 1.90) | 1.16 (0.86, 1.57) | 1.39 (1.04, 1.86) | -0.42 (0.41) |
| Bachelor’s degree | 1.26 (0.98, 1.62) | 1.03 (0.78, 1.36) | 1.26 (0.97, 1.65) | -0.06 (0.34) |
| Marital status (ref: single, never married) |  |  |  |  |
| Married | 1.25 (1.13, 1.39) | 1.20 (1.06, 1.35) | 1.25 (1.12, 1.40) | -0.50 (0.19) |
| Previously married | 1.39 (1.21, 1.61) | 1.22 (1.04, 1.44) | 1.45 (1.25, 1.68) | -0.48 (0.27) |
| Panel (ref: 1) |  |  |  |  |
| 2 | 1.39 (1.19, 1.62) | 1.35 (1.13, 1.60) | 1.53 (1.30, 1.81) | -1.43 (0.27) |
| 3 | 1.30 (1.12, 1.51) | 1.23 (1.04, 1.45) | 1.52 (1.29, 1.78) | -1.30 (0.26) |
| 4 | 1.50 (1.27, 1.78) | 1.90 (1.58, 2.28) | 1.75 (1.46, 2.09) | -3.86 (0.32) |
| **Military Characteristics at Time 1** | | | | |
| Military rank (ref: Officer) |  |  |  |  |
| Junior enlisted | 1.41 (1.12, 1.77) | 1.40 (1.09, 1.81) | 1.30 (1.03, 1.66) | -0.40 (0.36) |
| Noncommissioned officer | 1.32 (1.07, 1.64) | 1.34 (1.05, 1.71) | 1.23 (0.98, 1.55) | -0.57 (0.33) |
| Service branch (ref: Army) |  |  |  |  |
| Navy or Coast Guard | 0.71 (0.63, 0.80) | 0.73 (0.64, 0.84) | 0.75 (0.66, 0.86) | 1.14 (0.21) |
| Marine Corps | 1.01 (0.88, 1.15) | 0.90 (0.78, 1.05) | 0.97 (0.84, 1.11) | 0.08 (0.25) |
| Air Force | 0.66 (0.58, 0.74) | 0.75 (0.66, 0.86) | 0.76 (0.67, 0.86) | 1.00 (0.20) |
| Deployment experience (ref: not deployed) |  |  |  |  |
| Deployed, no combat | 1.05 (0.93, 1.19) | 1.00 (0.87, 1.15) | 0.98 (0.86, 1.12) | -0.36 (0.20) |
| Deployed with combat | 1.55 (1.40, 1.72) | 1.33 (1.19, 1.50) | 1.45 (1.30, 1.62) | -1.39 (0.19) |
| Military occupation (ref: other) |  |  |  |  |
| Admin or supply | 1.09 (0.99, 1.22) | 1.14 (1.01, 1.28) | 1.15 (1.03, 1.29) | -0.23 (0.18) |
| Healthcare | 1.23 (1.06, 1.43) | 1.16 (0.99, 1.37) | 1.17 (1.00, 1.37) | -0.91 (0.26) |
| Combat specialist | 1.26 (1.11, 1.42) | 1.09 (0.95, 1.25) | 1.15 (1.00, 1.31) | -0.55 (0.22) |
| Discharge condition (ref: honorable) |  |  |  |  |
| General, honorable conditions | 2.29 (1.75, 2.98) | 1.26 (0.93, 1.72) | 1.69 (1.28, 2.25) | -2.96 (0.59) |
| Bad, dishonorable, or other than honorable | 3.02 (2.08, 4.39) | 2.64 (1.76, 3.97) | 2.82 (1.91, 4.17) | -2.81 (0.84) |
| Reservist or National Guardsmen | 0.76 (0.68, 0.86) | 0.70 (0.61, 0.80) | 0.64 (0.56, 0.73) | 1.36 (0.20) |
| Years separated from military service | 1.00 (0.98, 1.03) | 0.98 (0.96, 1.01) | 1.01 (0.98, 1.03) | -0.02 (0.04) |
| **Behavioral/Physical/Mental Health at Time 1** | | | | |
| Problem drinking (ref: no) | 1.20 (1.07, 1.35) | 1.23 (1.08, 1.40) | 1.17 (1.03, 1.33) | -0.35 (0.23) |
| Smoking status (ref: never smoked) |  |  |  |  |
| Former smoker | 1.21 (1.09, 1.34) | 1.17 (1.04, 1.31) | 1.33 (1.19, 1.48) | -0.98 (0.18) |
| Current smoker | 1.27 (1.14, 1.42) | 1.30 (1.15, 1.47) | 1.45 (1.29, 1.63) | -1.28 (0.21) |
| Not recommended sleep duration (ref: 7-9 hours) | 1.74 (1.57, 1.92) | 1.64 (1.46, 1.84) | 1.60 (1.43, 1.78) | -0.95 (0.16) |
| Physical QOL | 0.97 (0.97, 0.98) | 0.98 (0.97, 0.98) | 0.98 (0.97, 0.98) | 0.15 (0.01) |
| Probable PTSD (ref: no) | 5.89 (5.27, 6.59) | - | - | - |
| Probable depression (ref: no) | - | 5.11 (4.50, 5.82) | - | - |
| Probable anxiety (ref: no) | - | - | 5.28 (4.66, 5.98) | - |
| Mental QOL | - | - | - | 0.49 (0.01) |
|  |  |  |  |  |

PTSD, posttraumatic stress disorder; QOL, quality of life; VHA, Veterans Health Administration. Models also included rurality, VHA utilization, Social Vulnerability Index, and distance from VHA facilities, with these results shown in Table 2. Unless otherwise noted, variables were significantly associated with mental health outcomes (*p* < .05) with the following exceptions. Probable PTSD: sex (*p*=.34), race and ethnicity (*p*=.07), and years since military separation (*p*=.76). Probable depression: sex (*p*=.15), race and ethnicity (*p*=.07), education (*p*=.75), military occupation (*p*=.10), and years since military separation (*p*=.14). Probable anxiety: pay grade (*p*=.10), and years since military separation (*p*=.69). Mental QOL: race and ethnicity (*p*=.09), education (*p*=.58), pay grade (*p*=.21), years since military separation (*p*=.68), and problem drinking (*p*=.13).
