# Supplementary material for: Rural residence and mental health among US Veterans: Findings from the Millennium Cohort Study
Source: PLoS One. 2026 Apr 17;21(4):e0346780. doi: 10.1371/journal.pone.0346780 (PMC13089753; doi:10.1371/journal.pone.0346780)
Supplement: S2 Table — (DOCX) [file pone.0346780.s002.docx]

**Rural residence and mental health among US Veterans:**

**Findings from the Millennium Cohort Study**

Claire A. Kolaja, Javier Villalobos, Julia Seay, Hope S. McMaster, Edward J. Boyko, Rudolph P. Rull, for the Millennium Cohort Study Team

PLOS One

**Definition of Rurality**

To supplement our main operationalization of rurality (3-level rurality using RUCA codes), we used the Index of Relative Rurality (IRR) – continuous measure of rurality – to characterize residential location at Time 2. This measure combines four indicators of rurality: population size, population density, remoteness, and built-up area (Waldorf, 2006; Waldorf and Kim, 2015; Kim and Waldorf, 2023). Values range from 0 to 1, with higher values representing more rural. In this sample, IRR ranged from 0.04 to 0.76 with an average IRR of 0.35 (standard deviation=0.11).

Supplemental Table 2 reports the bivariate and adjusted association between IRR and the mental health outcomes examined. At the bivariate level, a one unit change in rurality was associated with higher odds of screening positive for probable PTSD (OR=1.74; 95% CI=1.25, 2.43; *p-value*=0.001), depression (OR=1.57; 95% CI=1.06, 2.32; *p-value*=0.02), anxiety (OR=2.15; 95% CI=1.49, 3.11; *p-value*<0.0001), but not mental quality of life (*p-value*=0.51). In the fully adjusted models, IRR was not associated with probable PTSD (*p-value*=0.78), depression (*p-value*=0.39), or anxiety (*p-value*=0.98) but was significantly associated with mental quality of life (*p-value*=0.03). A one unit increase in rurality was associated with a 1.54 (standard error: 0.71) unit higher mental quality of life. The interactions between IRR and VHA utilization did not reach statistical significance (*p-value* > 0.05) for any of the outcomes examined.

| **Supplemental Table 2: Associations between continuous rurality and mental health outcomes, n=20,418** | | |
| --- | --- | --- |
|  | **Bivariate** | **Adjusted**± |
|  | **Probable PTSD**  **OR (95% CI)** | |
| Rurality (continuous, 1-unit increase) | 1.74 (1.25, 2.43)* | 0.94 (0.63, 1.42) |
|  | **Probable Depression**  **OR (95% CI)** | |
| Rurality (continuous, 1-unit increase) | 1.57 (1.06, 2.32)* | 0.82 (0.52, 1.29) |
|  | **Probable Anxiety**  **OR (95% CI)** | |
| Rurality (continuous, 1-unit increase) | 2.15 (1.49, 3.11)* | 1.01 (0.65, 1.55) |
|  | **Mental QOL**  **Coefficient (SE)** | |
| Rurality (continuous, 1-unit increase) | -0.53 (0.81) | 1.54 (0.71)* |
| **p-value* < 0.05; ±Models adjusted for panel, age, sex, race and ethnicity, education attainment, marital status, paygrade, service branch, military occupation, deployment experience, discharge status, problem drinking, smoking status, physical components summary score, mental health, sleep duration, years since separation, social vulnerability, drive time from VHA facilities, and VHA utilization. | | |

**References:**

Waldorf, Brigitte S. "A continuous multi-dimensional measure of rurality: Moving beyond threshold measures." (2006). Accessed 1/06/2026 at <https://ageconsearch.umn.edu/record/21383?v=pdf>.

Waldorf, Brigitte, and Ayoung Kim. " Defining and measuring rurality in the US: From typologies to continuous indices.” In *Commissioned paper presented at the Workshop on Rationalizing Rural Area Classifications, Washington, DC*. (2015): R7959FS8.

Kim, Ayoung, and Brigitte Waldorf. "The Index of Relative Rurality (IRR): US County Data for 2020." (2023). DOI: 10.5281/zenodo.7675745
